# Stimulatory Effects of Cinnamon Extract (*Cinnamomum cassia*) during the Initiation Stage of 3T3-L1 Adipocyte Differentiation

**DOI:** 10.3390/foods5040083

**Published:** 2016-12-06

**Authors:** Sang Gil Lee, Joanna A. Siaw, Hye Won Kang

**Affiliations:** Food and Nutritional Sciences, Department of Family and Consumer Sciences, North Carolina Agricultural and Technical State University, 1601 E. Market Street, Greensboro, NC 27411, USA; slee123@ncat.edu (S.G.L.); josiaw5@gmail.com (J.A.S.)

**Keywords:** cinnamon, white adipocyte differentiation, fatty acid oxidation, lipogenesis, adipogenesis

## Abstract

Cinnamon (*Cinnamomum cassia*) has an anti-diabetic effect by possibly increasing the lipid storage capacity of white adipocytes; however, this effect remains controversial. The aim of this study was to examine which stage of adipogenesis is critical for the stimulatory effect of cinnamon in adipogenesis using 3T3-L1 cells. Cells were treated with cinnamon extract during three different stages of adipogenesis. We found that genes related to adipogenesis and lipogenesis were enhanced when cinnamon extract was administered during the initiation stage of differentiation but not when administered during the preadipocyte and post stages of differentiation. At the same time, genes that were involved in the regulation of fatty acid oxidation were unexpectedly upregulated. Taken together, cinnamon may boost lipid storage in white adipocytes and increase the fatty acid oxidation capacity throughout the initiation stage of differentiation, which may be beneficial for the prevention of obesity-induced type II diabetes.

## 1. Introduction

White adipose tissue (WAT) is the primary site where extra calories are stored as fat for the body’s energy reserve. WAT also secretes several hormones and cytokines that are critical for regulating nutrient metabolism [1]. However, excessive fat accumulation in WAT causes obesity and adipocyte dysfunction [2], resulting in a spillover of fatty acids into non-adipose organs, which further increases susceptibility for the development of type 2 diabetes mellitus, insulin resistance, and dyslipidemia [3]. Therefore, it is important for WAT to maintain its normal function and capacity to safely store fat, which protects other tissues against lipotoxicity [4]. This maintenance can be achieved by acquiring new adipocytes through adipocyte differentiation in WAT. Adipocyte differentiation is the process in which preadipocytes develop into mature white adipocytes that accumulate lipids as a single lipid droplet through early, intermediate, and terminal stages [5].

Various transcriptional factors and enzymes are involved in the adipogenesis process. Peroxisome proliferator-activated receptor gamma (PPARγ) is a primary transcriptional factor that regulates adipogenesis and also operates as a known therapeutic target for type 2 diabetes mellitus and dyslipidemia to increase the lipid storage capacity of WAT [6]. PPARγ agonists, such as thiazolidinediones (TZDs), increase the WAT storage capacity by activating PPARγ, subsequently improving insulin sensitivity [7]. However, various side effects of these drugs have demanded the development of alternative therapeutics from natural sources that activate PPARγ [6,8]. Cinnamon, the bark of *Cinnamomum cassia*, has been used extensively as a traditional herb to manage numerous health conditions and as a spice in the food industry [9]. Previous studies have shown that cinnamon extract (CE) and cinnamaldehyde, a bioactive compound of cinnamon, improved insulin sensitivity and reduced plasma glucose levels, possibly via PPARγ [10,11,12].

However, some discrepancies remain regarding the anti-diabetic effect of cinnamon and whether cinnamon affects the fat storage capacity of adipocytes through PPARγ activation. Type II diabetic patients who took capsules containing cinnamon powder during a two-month period did not exhibit improvement in either plasma glucose or HbA1c levels [13]. In addition, Han and Huang et al. showed an inhibitory effect of cinnamon during adipogenesis [14,15]. Therefore, the aim of this study was to investigate the effects of CE in the three different stages of adipogenesis that regulate the fat storage capacity of adipocytes. These effects were defined by examining the expression profiles of genes related to adipogenesis, lipogenesis, and fatty acid oxidation in adipocytes that were fully differentiated and matured after CE was administered during the three different stages of adipogenesis. 

## 2. Materials and Methods

### 2.1. Sample Preparation

CE (from *Cinnamomum cassia*) was purchased from New Age Botanicals (Melbourne, Australia). CE powder was diluted in culture media to final concentrations of 50, 100, and 200 μg/mL with 0.1% dimethyl sulfoxide (DMSO). The sample solution was then filtered using a syringe filter (0.22 μM). The nutritional value and phenolic content of CE powder are shown in the Supplementary Table S1.

### 2.2. Cell Culture

**Figure 1:**
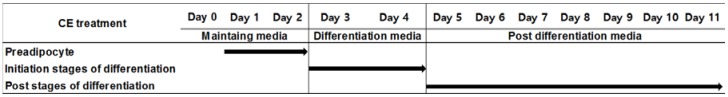
Experimental design to investigate the stimulatory effects of cinnamon extract (CE) treatments at different stages of 3T3-L1 adipocyte adipogenesis. 3T3-L1 adipocytes were treated with CE at the preadipocyte (days 1–2), initiation (days 3–4), and post stages (days 5–11) of differentiation during adipogenesis. When adipocytes were fully differentiated and matured on day 11, cells were harvested to extract total RNA for measuring gene expression using quantitative real-time PCR and were stained with Oil-Red O to visualize lipid accumulation as a marker of differentiation.

3T3-L1 cells were purchased from the American Type Culture Collection (ATCC, Manassas, VA, USA) and were maintained in Dulbecco’s Modified Eagle’s Medium (DMEM) supplemented with 10% bovine calf serum in a humidified cell culture incubator (37 °C and 5% CO_2_). For experiments, cells were seeded at a density of 17,500 cells/cm^2^ in 12-well plates with DMEM supplemented with 10% fetal bovine serum (FBS), indicated as day 0. On day 3, the culture medium was switched to differentiation medium (DMEM, 10% FBS, 1 μg/mL insulin, 0.5 mM 3-isobutyl-1-methylxanthine, and 0.25 mM dexamethasone), and the cultures were incubated for two additional days. On day 5, the differentiation medium was replaced with post differentiation medium (DMEM, 10% FBS, and 1 μg/mL insulin). The post differentiation medium was refreshed every other day until day 11. As shown in Figure 1, to determine the effects of CE on preadipocytes, the initiation of differentiationand its progress during the differentiation and maturation process, cells were treated with CE at concentrations of 50, 100, and 200 μg/mL on day 1 (preadipocyte stage), day 3 (initiation stage of differentiation) or days 5, 7, and 9 (post stages of differentiation) and were incubated for two additional days. The cells were then cultured and differentiated as described above until day 11. On day 11, cells were collected for total RNA extraction and Oil-Red O staining.

### 2.3. Cell Viability Assay

Cell viability was determined using a thiazolyl blue tetrazolium bromide (MTT) reduction assay according to the manufacturer’s instructions (Cayman, Ann Arbor, MI, USA). Briefly, 3T3-L1 preadipocytes were seeded in a 96-well plate at a density of 17,500 cells/cm^2^. The next day, the culture medium was replaced with fresh medium containing various concentrations of CE (0–1000 µg/mL). After 48 h of incubation, the media were replaced again with fresh culture medium containing 0.5 mg/mL MTT reagent, and cells were incubated for 3 h at 37 °C. Afterwards, the medium was discarded, and 100 μL of dimethyl sulfoxide (DMSO) was added to solubilize the purple formazan products. The absorbance was determined at 570 nm using a Synergy HT Microplate Reader (BioTek, Winooski, VT, USA), which indicates proportionally the number of viable cells. Cell viability was expressed as a percentage of the absorbance of cells treated with CE relative to the absorbance of cells that were not treated with CE. Experiments were performed in triplicate.

### 2.4. Quantitative Real-Time Polymerase Chain Reaction (qPCR)

Total RNA was extracted from fully differentiated adipocytes (day 11) using TRIzol (Thermo Fisher Scientific, Waltham, MA, USA) according to the manufacturer’s instructions. RNA concentrations and purity were determined using a Take3 micro-volume plate equipped with a Synergy HT microplate reader (BioTek). RNA was transcribed to complementary DNA (cDNA) using XLAScript cDNA MasterMix (Exella GmbH, Feucht, Germany) according to the manufacturer’s instructions. To determine the expression of target genes, first-strand cDNA was amplified using a Fast Start Essential DNA Green Light Master kit (Roche, Indianapolis, IN, USA) in a Light Cycler 90 (Roche). PCR conditions were as follows: 10 min at 95 °C, followed by 50 cycles of 10 s denaturation at 95 °C, annealing for 10 s at 60 °C, and extension for 10 s at 72 °C. The primers used are shown in Table 1. Ribosomal protein L 32 (RPL32) was used as a housekeeping gene. Cycle threshold (*C*_t_) values were obtained. The expression level of each gene was calculated using the 2 delta *C*_t_ method by normalizing the *C*_t_ value of the targeted gene to that of the RPL32 gene. The data are presented as relative percentages of the expression level of each target gene when gene expression levels in adipocytes that were not treated with CE (control) were 100%.

### 2.5. Oil-Red O Staining

Intracellular lipid accumulation was determined using Oil-Red O staining (Thermo Fisher Scientific). Differentiated adipocytes (day 11) were rinsed with phosphate-buffered saline (PBS) twice and were fixed in 10% buffered formalin for 1 h at room temperature. After rinsing with 60% isopropanol, cells were incubated with 60% isopropanol-based Oil-Red Solution by mixing 2 parts of deionized (DI) water and 3 parts of stock solution (350 mg of Oil-Red O in 100 mL of isopropanol) for 30 min at room temperature. Oil-Red O, an oil-soluble dye used to stain lipids, was removed and washed twice with DI water. Lipid droplets were visualized based on their red color using an Evos XL-microscope (Thermo Fisher Scientific).

### 2.6. Data Analysis

The data are presented as means ± standard error of mean (SEM). The comparisons were analyzed using one-way analysis of variance (ANOVA) with Tukey’s post-hoc test in Prism 6 (GraphPad Software Inc., La Jolla, CA, USA). All differences were considered significant at *p* < 0.05.

## 3. Results

### 3.1. Cytotoxicity of CE in Preadipocytes

**Figure 2:**
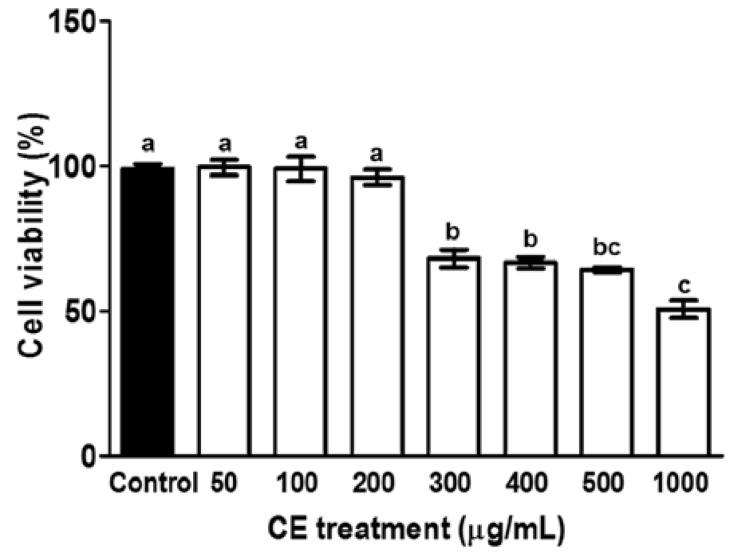
Cytotoxicity of CE in 3T3-L1 preadipocytes. Preadipocytes were treated with different concentrations of CE for 48 h. Cell viability was then measured using a thiazolyl blue tetrazolium bromide (MTT) reduction assay. The experiment was performed in triplicate. A different letter indicates a statistically significant difference (*p* < 0.05).

CE did not induce cytotoxicity at or below 200 µg/mL (Figure 2). Thus, the concentration ranges of CE selected for treatment in 3T3-L1 cells were 50, 100, and 200 µg/mL.

### 3.2. CE Increased Lipid Accumulation by Increasing the Expression of Adipogenic and Lipogenic Genes during the Initiation Stage of Differentiation

**Figure 3:**
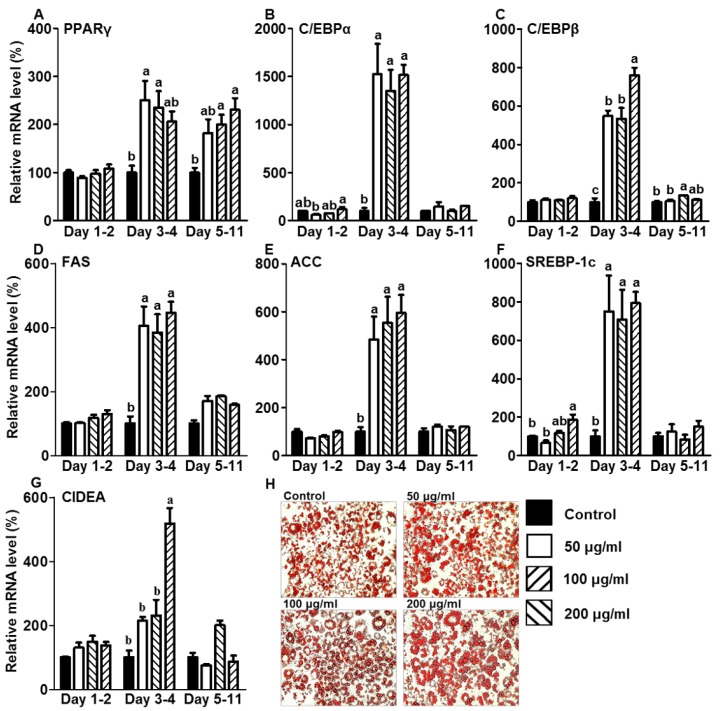
Effects of CE treatment at different stages of 3T3-L1 adipogenesis on adipogenic and lipogenic gene expression levels. Adipocytes were treated with CE at the preadipocyte (days 1–2), initiation (days 3–4), and post stages (days 5–11) of differentiation during adipogenesis. On day 11, the expression levels of genes related to adipogenesis (**A**) PPARγ, (**B**) C/EBPα, (**C**) C/EBPβ, and lipogenesis (**D**) FAS, (**E**) ACC and (**F**) SREBP-1c, and lipid droplet formation (**G**) CIDEA, were measured using a quantitative real-time PCR assay. (**H**) Representative images of Oil-Red O staining in fully matured adipocytes after adipocytes were treated with CE during the initiation stage of differentiation. The experiment was performed in triplicate. The statistical differences among the four CE concentrations (0, 50, 100, and 200 μg/mL) that were used to treat cells were separately analyzed for each experiment and each time window (e.g., day 1–2, day 3–4, and day 5–11) using one-way ANOVA with Tukey’s post hoc test. A different letter indicates a statistically significant difference (*p* < 0.05).

As shown in Figure 3A, CE treatment during the initiation or post stages of differentiation upregulated PPARγ gene expression during the adipogenesis process. PPARγ coordinately works with CCAAT/enhancer-binding protein alpha and beta (C/EBPα and β) to mediate a differentiation process that converts preadipocytes into mature adipocytes [16]. Figure 3B,C show that the C/EBPα and β genes were upregulated in adipocytes treated with CE in the initiation stage of differentiation during adipogenesis. Although C/EBPβ gene expression was increased slightly in adipocytes when CE was administered during the post stages of differentiation, there was no change in C/EBPα expression (Figure 3B,C). CE treatment during the preadipocyte stage did not alter the mRNA expression levels for any of the three genes related to adipogenesis, PPARγ, C/EBPα, or β, during adipogenesis (Figure 3A–C). Consistent with increased expression of genes that are involved in the regulation of adipogenesis, fatty acid synthase (FAS), acetyl-CoA carboxylase (ACC), and sterol regulatory element-binding protein 1c (SREBP-1c) genes, also involved in lipogenesis, were affected in adipocytes treated with CE during the different stages of adipogenesis (Figure 3D–F). The addition of CE in the preadipocyte stages barely affected the expression levels of FAS, ACC, or SREBP-1c genes when the cells became fully mature, while CE treatment at 200 µg/mL exhibited an increase in SREBP-1c gene expression. Adipocytes treated with CE during the initiation stage of differentiation increased SREBP-1c, FAS, and ACC mRNA expression levels compared with control cells when they became mature adipocytes. CE treatment during the post differentiation stage did not affect FAS, ACC, or SREBP-1c gene expression. As shown in Figure 3G, the cell death-inducing DFFA-like effector (CIDEA) gene, which is known to play a role in the formation of lipid droplets, was also upregulated in mature adipocytes following treatment with 200 µg/mL CE during the initiation stage of differentiation. Figure 3H shows the increased lipid accumulation in mature adipocytes following treatment with CE during the initiation stage of differentiation.

### 3.3. CE Elevated the mRNA Expression Levels of Genes Related to Fatty Acid Oxidation in the Initiation Stage of Differentiation during Adipogenesis

**Figure 4:**
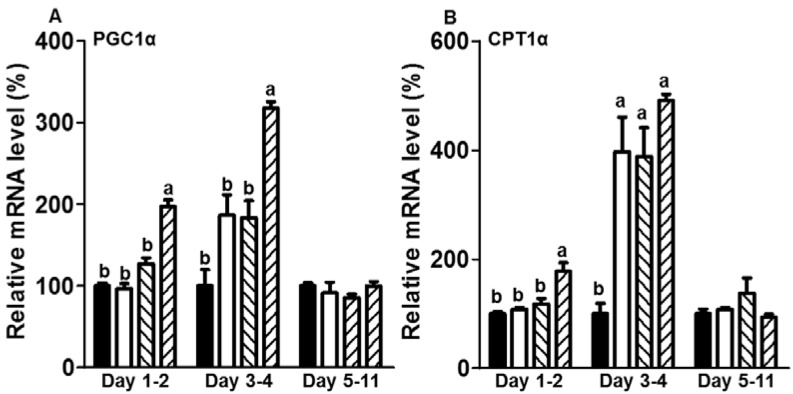
Effects of CE treatment at the different stages of 3T3-L1 adipogenesis on genes related to fatty acid oxidation. Adipocytes were treated with CE at the preadipocyte (days 1–2), initiation (days 3–4), and post stages (days 5–11) of differentiation during adipogenesis. On day 11, the expression levels of genes related to fatty acid oxidation (**A**) PGC1α and (**B**) CPT1α were measured using a quantitative real-time PCR assay. The experiment was performed in triplicate. The statistical differences among the four CE concentrations (0, 50, 100, and 200 μg/mL) that were used to treat cells were separately analyzed for each experiment and each time window (e.g., day 1–2, day 3–4, and day 5–11) using one-way ANOVA with Tukey’s post-hoc test. A different letter indicates a statistically significant difference (*p* < 0.05).

Figure 4A,B show the effects of CE on the expression levels of genes related to the regulation of fatty acid oxidation. When preadipocytes were treated with 200 µg/mL CE, the expression of the PPARγ-coactivator 1 alpha (PGC1α) gene, which encodes a transcriptional factor to regulate energy metabolism including fatty acid oxidation, was increased in fully differentiated mature adipocytes after adipogenesis (Figure 4A). PGC1α gene induction was also observed in adipocytes treated with 200 µg/mL CE during the initiation stage of differentiation. However, PGC1α gene expression was not changed by CE treatment during the post differentiation stages (Figure 4A). Consistent with the stimulatory effect of CE treatment on the PGC1α gene at the preadipocyte and initiation stages of differentiation, treatment with 200 µg/mL CE during the initiation stage of differentiation increased mRNA expression levels of the carnitine palmitoyltransferase (CPT) 1α gene, which is involved in promoting fatty acid oxidation during adipogenesis (Figure 4B). However, there were no changes in CPT1α gene expression when preadipocytes were treated with concentrations of CE below 200 µg/mL. Treatment with CE at concentrations of 50, 100, and 200 µg/mL during the initiation stage of differentiation strongly elevated CPT1α mRNA levels when adipocytes were fully differentiated. Treatment with CE during the post differentiation stages did not affect CPT1α gene expression during adipogenesis (Figure 4B).

## 4. Discussion

Increasing the lipid storage capacity of WAT with anti-diabetic medicines, such as TZDs, can reduce the development of type 2 diabetes by lowering circulating fatty acid and triglyceride levels [6,17]. Cinnamon is a functional food that may improve the lipid loading capacity of WAT by enhancing PPARγ activity [10,18]. However, the stimulatory effects of cinnamon on PPARγ remain controversial. It is also unclear which adipogenic stage is critical for the stimulatory effect of cinnamon to increase the capacity of white adipocytes to store lipids. In the present study, we found that CE increased the lipid storage capacity during the initiation stage of differentiation by upregulating the expression levels of genes related to adipogenesis and lipogenesis. Additionally, CE showed potential for increasing fatty acid oxidation during adipogenesis, specifically as a result of CE treatment during the initiation stage of differentiation. Therefore, our present study demonstrated that the initiation of differentiation is a critical stage for cinnamon-stimulated effects in adipogenesis and lipogenesis to increase the lipid storage capacity in white adipocytes, as confirmed by increased lipid accumulation as a marker of promoted differentiation. We also observed that CE treatment during the initiation stage of differentiation could induce adipocytes to perform dual functions of lipid storage and utilization.

WAT contains adipocytes that are in various stages of development. For adipocytes to obtain the capacity to store lipids, a process called adipogenesis is required to differentiate preadipocytes into mature adipocytes. Although adipogenesis is regulated by coordinating with various transcriptional factors, e.g., PPARγ, C/EBPα, C/EBPβ, and adipocyte protein 2 (aP2), PPARγ is recognized as a primary regulator for adipogenesis [16]. C/EBPβ is induced at the early stage of differentiation and increases the activity of PPARγ and C/EBPα throughout the later stages of differentiation to promote differentiation, which further increases the utility of aP2 as a terminal marker of differentiation [16]. Consistent with the increased PPARγ, C/EBPα, and C/EBPβ gene expression levels noted in our present study, Sheng at al. observed similar stimulatory effects of CE during the induction of differentiation in 3T3-L1 cells by increasing PPARγ expression and transcriptional activity at a concentration of 600 µg/mL, which was cytotoxic in our present study [18]. In contrast, one study showed that 100 and 500 µg/mL CE inhibited adipogenesis and lipogenesis by decreasing PPARγ gene expression [14]. Another study also reported that cinnamaldehyde, one of the active compounds found in cinnamon, prevented PPARγ transcriptional activity [15]. It has been reported that cinnamon includes various dietary compounds, such as coumarin, 2-hydroxyl cinnamaldehyde, cinnamyl alcohol, cinnamic acid, cinnamaldehyde, 2-methoxy cinnamaldehyde, and eugenol [14]. The differences in the effects of CE between studies may depend on different concentrations of other major dietary compounds as a result of the different extraction methods used. As shown in our present study, the treatment stage was critical for acquiring the different effects of CE.

Along with adipogenesis, lipogenesis is also promoted to store lipids as triglycerides in adipocytes during differentiation. Consistent with the increased expression levels of the PPARγ, C/EBPα, and C/EBPβ genes, the FAS, ACC, and SREBP-1c genes, which are involved in the regulation of lipogenesis, were also increased when CE was administered during the initiation of differentiation. The increases in both adipogenesis and lipogenesis in response to CE treatment during the initiation stage of differentiation were further confirmed by increased lipid accumulation and larger-sized lipid droplets. Mice treated with a PPARγ agonist, rosiglitazone, exhibited upregulated CIDEA expression in WAT [19]. CIDEA plays a role in the enlargement of lipid droplets by transferring triglycerides between neighbored lipid droplets [20]. Transgenic mice expressing human CIDEA became obese by expanding the sizes of their adipose tissues, but had increased insulin sensitivity [21]. In contrast, CIDEA deficient-mice were lean and had small lipid droplets [22]. In the present study, the larger-sized lipid droplets produced in response to CE treatment during the initiation stage of differentiation may have resulted from increased expression of the CIDEA gene. It has also been reported that CIDEA expression was induced by PGC1α [23]. PGC1α is a positive regulator of catabolic metabolism, e.g., fatty acid oxidation, and is abundantly present in oxidative tissues, such as brown adipose tissue, muscle, and liver. Our present study showed that CE elevated PGC1α mRNA levels in adipocytes at the initiation stage of differentiation. Therefore, CE treatment during the initiation stage of differentiation may have increased CIDEA expression by activating PGC1α and PPARγ.

Although the fat storage capacity of WAT may positively improve diabetic conditions, expanding the size of WAT by accumulating excessive fat also presents some concern, e.g., obesity. However, the present study indicated that CE may improve mitochondrial fatty acid oxidation in the initiation of differentiation, likely in part due to the increased expression of CPT1α and PGC1α genes. C/EBPβ is involved in not only adipogenesis but also energy metabolism [24]. When C/EBPβ was transiently overexpressed in 3T3-L1 cells, the cells displayed brown-like characteristics by increasing PGC1α expression, which is indicated as the browning of white adipocytes [24]. Therefore, increased expression of fatty acid oxidation genes, including CPT1α and PGC1α in response to treatment with CE at the initiation stage of differentiation, would be related to C/EBPβ upregulation. 

## 5. Conclusions

In conclusion, the present study demonstrated that CE allows adipocytes to have enhanced lipid storage capacity and fatty acid oxidation in the initiation stage of differentiation during adipogenesis by simultaneously increasing the expression levels of genes related to adipogenesis, lipogenesis, and fatty acid oxidation. It is possible that the dual effects of CE on both the storage and utilization of fat would be beneficial for obesity related to type II diabetes by increasing insulin sensitivity through enhanced lipid storage capacity and by utilizing stored fat to avoid excess fat accumulation. Although further studies using animal models to understand the effect and efficiency of CE in vivo should be conducted, our findings would provide meaningful information for the development of new anti-diabetic drugs that act on specific target stages of adipogenesis. 

## Figures and Tables

**Table 1:** Primers designed for quantitative real-time PCR.

Gene	Forward Primer	Reverse Primer
ACC	TGCATTCTGACCTTCACGAC	ACATCCACTTCCACACACGA
C/EBPα	GGACAAGAACAGCAACGAGTA	GCAGTTGCCATGGCCTTGA
C/EBPβ	TGGACAAGCTGAGCGACGAG	TGTGCTGCGTCTCCAGGTTG
CIDEA	ATCACAACTGGCCTGGTTACG	TACTACCCGGTGTCCATTTCT
CPT1α	TTTGACTTTGAGAAATACCCTGATA	TGGATGAAATTCTCTCCCACAATAA
FAS	TGGGTTCTAGCCAGCAGAGT	ACCACCAGAGACCGTTATGC
PGC1α	TGCCCAGATCTTCCTGAACT	TCTGTGAGAACCGCTAGCAA
PPARγ	TTTGACTTTGAGAAATACCC	TGGATGAAATTCTCTCCAC
RPL32	CACCAGTCAGACCGATAT	TTCTCCGCACCCTGTTG
SREBP-1c	GAACAGACACTGGCCGAGAT	GAGGCCAGAGAAGCAGAAGAG

Abbreviations: ACC, acetyl-CoA carboxylase; C/EBPα, CCAAT-enhancer-binding protein α; C/EBPβ, CCAAT-enhancer-binding protein β; CIDEA, cell death-inducing DFFA-like effector; CPT1α, carnitine palmitoyltransferase 1α; FAS, fatty acid synthase; PGC1α, peroxisome proliferator-activated receptor gamma coactivator 1α; PPARγ, peroxisome proliferator-activated receptor gamma; RPL32, ribosomal protein L 32; SREBP-1c, sterol regulatory element-binding protein 1.

## Supplementary Materials

The following are available online at www.mdpi.com/2304-8158/5/4/83/s1, Table S1: *F*-values in the statistical analysis for gene expressions level by cinnamon extract (CE) treatments, Table S2: Nutritional components of 100 g cinnamon extract (CE) powder.
