# Supplementary material for: Stimulatory Effects of Cinnamon Extract (Cinnamomum cassia) during the Initiation Stage of 3T3-L1 Adipocyte Differentiation
Source: Foods. 2016 Dec 6;5(4):83. doi: 10.3390/foods5040083 (PMC5302429; doi:10.3390/foods5040083)
Supplement: Supplementary file 1 [file foods-05-00083-s001.pdf]

Supplementary Materials: Stimulatory Effects of Cinnamon Extract (*Cinnamomum cassia*) during the Initiation Stage of 3T3-L1 Adipocyte Differentiation

Sang Gil Lee, Joanna A. Siaw and Hye Won Kang

Table S1. *F*-values in the statistical analysis for gene expressions level by cinnamon extract (CE) treatments [1].

	CE Treatment Stage		
**Genes**	**Pre-Adipocyte**	**Initiation**	**Adipocyte**
PPARγ	1.47	5.34	6.54
CEBPα	4.89	11.96	1.49
CEBPβ	1.14	53.58	8.06
SREBP1c	10.45	6.27	1.04
FAS	2.87	11.58	14.04
ACC	4.41	7.60	0.89
CPT1α	13.34	15.96	1.68
UCP1	3.47	0.15	7.15
PRDM16	6.28	0.08	6.37

[1] Degrees of freedom in the analysis is 3.

Table S2. Nutritional components of 100 g cinnamon extract (CE) powder.

Component	Amount (g)
Protein	11
Fat Acid	5
Carbohydrates	12
Cellulose	13
Vitamin E	7
Manganese	9
Potassium	11
Calcium	15
Phosphorus	1
Sodium	1
Selenium	1
Iron	1
Water	3
Total phenolics [1]	14.1

[1] Total phenolic amounts were measured by the Folin-Ciocalteu method and expressed as g gallic acid equivalent/100 g of CE powder.
